# Advances in Molecular Biomarkers in Cardiology

**DOI:** 10.3390/biom12101530

**Published:** 2022-10-21

**Authors:** Pietro Scicchitano, Matteo Cameli

**Affiliations:** 1 Cardiology Section, Hospital “F. Perinei” ASL BA, 70022 Altamura, Bari, Italy; 2 Department of Medical Biotechnologies, Division of Cardiology, University of Siena, 53100 Siena, Italy

Cardiovascular diseases (CVD) represent the leading cause of death in the world despite innovations in therapies and advances in the general management of patients [1].

Early diagnosis and accurate risk stratification are the mainstays for preventing adverse events or, at least, reducing the occurrence of the worst consequences related to CVD.

Biomarkers—identified by the U.S. Food and Drug Administration as “a defined characteristic that is measured as an indicator of normal biological processes, pathogenic processes or responses to an exposure or intervention” [2]—are one of the most intriguing and challenging fields of research during the last decades, especially within cardiology setting.

The need for early diagnosis of diseases, surveillance and clinical monitoring of patients, management of therapeutic variations, and prognostic risk stratification lead physicians to consider several biomarkers in clinical practice.

Indeed, the challenge in the development of the perfect cardiovascular (CV) biomarkers is the identification of a substance able to be safe and easy to measure, cost-effective, highly reproducible, able to accordingly vary across the spectrum of the disease, and independent from gender/ethnicity and/or other characteristics [3].

Research is ongoing for overcoming the main limitations of currently available biomarkers—namely, the lack of biomarkers for specific diseases, costs for the identification of ideal biomarkers, and reproducibility of the identified molecule, parameter, etc. [3].

This Special Issue entitled Special Issue “Molecular Biomarkers In Cardiology 2021” tried to provide novel insights into the general landscape of biomarkers in the setting of CVD prediction, evaluation, and management.

One of the most intriguing issues is the need for reproducibility. Ain et al. [4] described the influence of several conditions such as age, sex, socio-economic status, body mass index, medication and other substance use, and medical illness, as well as inconsistencies in methodological practices such as sample collection, assaying, and data cleaning and transformation when dealing with the reproducibility of biomarkers in CVD. Aguilar-Iglesias et al. [5] evaluated the impact of frailty and age on the performance of biomarkers in the diagnosis of acute heart failure (AHF). Frailty effectively impacts the performance of NT-proBNP in detecting AHF in patients older than 75 years old, while no influence was in younger [5]. Indeed, AHF might be considered a complex pathological entity derived from several conditions. Nawrocka-Millward et al. [6] observed differences in biomarkers prognostic performance of biomarkers—namely serum lactate, bilirubin, matrix metallopeptidase 9, follistatin, intercellular adhesion molecule 1, lipocalin, and galectin-3—in patients with different types of AHF.

Indeed, biomarkers might act as predictors in several types of diseases. Merino-Merino et al. [7] summarized the role of galectin-3 and the suppressor of tumorigenicity-2 (ST2) in patients with coronary artery disease (CAD) and/or atrial fibrillation, although these two molecules have been widely adopted as biomarkers in HF. Osteopontin might effectively act as an inflammatory biomarker in CVD as its chronic increase might be related to adverse events [8].

Biomarkers should ameliorate risk stratification in the general population. Conditions such as diabetes and thyroid diseases might impact cardiac health. Sirtuin-1 and interleukin-27 serum concentration might increase in case of cardiac dysfunction in a patient with type-1 diabetes and Hashimoto’s disease, thus suggesting a possible role of both conditions on overall cardiac protection [9].

The need for monitoring patients with end-stage HF is still a matter of debate. Sciaccalunga et al. [10] provided a comprehensive analysis of biomarkers to be adopted for the clinical management and prognostic evaluation of patients with the Left Ventricular Assist Device (LVAD).

The era of precision medicine is promoting advancement in the diagnosis and prognosis of CVD. Elwazir et al. [11] proposed the use of specific angiogenesis-related long non-coding RNAs (lncRNAs) for the general assessment of individuals at risk for CAD or the identification of the severity of CAD. Indeed, the lncRNA variants were mostly able to predict the severity of CAD—as assessed by the Gensini score—rather than identifying disease susceptibility. Extracellular circulating microRNAs (miRNAs) might provide further insight into distinguishing between CAD and acute coronary syndromes (ACSs). Zhelankin et al. focused their attention on miR-21-5p, miR-146a-5p, and miR-17-5p in CAD and ACS patients [12]. No matter the type of ACSs, miR-21-5p and miR-146a-5p increased, while lower serum concentrations in miR-17-5p were found in those suffering from CAD. Nevertheless, transcriptomic and genetic variability might also impact the development of CAD. For instance, polymorphisms of interferon regulatory factor 5 might effectively alter the cardiometabolic profile of individuals, thus predisposing them to atherosclerosis and its consequences [13].

Nevertheless, despite the great efforts from the scientific community, the way for the identification of the perfect biomarkers of plaque instability is too far from being completed [14]. Genomics, transcriptomics, proteomics, and other common circulating biomarkers are not eventually able to identify the point-break of the atherosclerotic plaque: further research should be dedicated to this interesting and revolutionary issue [14].

Finally, this Special Issue also dealt with some peculiar aspects of CVD. The prognosis of patients with atrial ischemia (AI) is still a matter of debate. The literature is scant on this issue. Kacprzak et al. [15] demonstrated no role for N-terminal pro-atrial natriuretic peptide in predicting AI in patients with myocardial infarction, although it predicted major adverse cardiovascular events.

The worldwide impact of the SARS-CoV-2 pandemic forced physicians to better understand the pathogenesis of the disease in order to counteract adverse events. Hopp et al. [16] provided exciting research that mixed computational and clinical approaches for the evaluation of the role of heme in the pathogenesis of systemic lesions related to the COVID-19 infection.

Finally, an interesting overview of the mechanism and possible therapeutic target for the treatment of venous thromboembolism in patients with cancer has been performed by Canonico et al. [17]. Further insights should be provided by next-generation clinical trials in order to better understand the best approach for the management of these patients.

This Special Issue collected intriguing proposals for the diagnosis, management, and prognostic stratification of patients with CAD and HF. The authors tried to shed light on the field of biomarkers in CVD by promoting their innovative research dealing with molecular aspects and clinical perspectives. Further research is welcomed for improving knowledge of CVD and its prevention/management.
